# Specific Learning Disorders (SLD) and Behavior Impairment: Comorbidity or Specific Profile?

**DOI:** 10.3390/children10081356

**Published:** 2023-08-07

**Authors:** Daniela Pia Rosaria Chieffo, Valentina Arcangeli, Federica Moriconi, Angelica Marfoli, Federica Lino, Sofia Vannuccini, Elisa Marconi, Ida Turrini, Claudia Brogna, Chiara Veredice, Alessandro Antonietti, Gabriele Sani, Eugenio Maria Mercuri

**Affiliations:** 1 Clinical Psychology Unit, Fondazione Policlinico Universitario Agostino Gemelli IRCCS, 00168 Rome, Italy; valentina.arcangeli@policlinicogemelli.it (V.A.); federica.moriconi@guest.policlinicogemelli.it (F.M.); federica.lino@policlinicogemelli.it (F.L.); vannuccini.sofia@gmail.com (S.V.); elisa.marconi@policlinicogemelli.it (E.M.); 2 Department Women Children and Public Health, Catholic University of Sacred Heart, 00168 Rome, Italy; eugeniomaria.mercuri@policlinicogemelli.it; 3 Department of Pediatric Neurology, Catholic University, 00168 Rome, Italy; ida.turrini@guest.policlinicogemelli.it (I.T.); claudia.brogna@policlinicogemelli.it (C.B.); chiara.veredice@policlinicogemelli.it (C.V.); 4 Department of Psychology, Università Cattolica del Sacro Cuore, 20123 Milan, Italy; alessandro.antonietti@unicatt.it; 5 Institute of Psychiatry and Psychology, Department of Geriatrics, Neuroscience and Orthopedics, Fondazione Policlinico Universitario Agostino Gemelli IRCCS, Università Cattolica del Sacro Cuore, 00168 Rome, Italy; gabriele.sani@policlinicogemelli.it

**Keywords:** specific learning disorder, SLD, cognitive profile, working memory, literacy skills, behavior impairment

## Abstract

Introduction: Specific Learning Disorder (SLD) is a neurodevelopmental disorder characterized by difficulties in perceiving and processing verbal and non-verbal information. It is usually accompanied by impaired academic skills leading to school dropout and emotional disturbances, resulting in significant distress and behavioral problems. Methods: A cognitive, academic, and emotional-behavioral assessment was performed at T0 and T1 in children and adolescents with SLD. Participants received psychotherapy and speech therapy treatment from T0 to T1. Results: In SLD, the most compromised cognitive functions were working memory and writing skills. An impact on academic abilities was found. Children and adolescents with SLD experience greater anxiety and depression levels compared to their control peers. Conclusions: SLD may adversely influence psychological well-being. To counteract such a consequence, more specific cognitive and academic skill-oriented strategies should be taken into consideration.

## 1. Introduction

Specific Learning Disorder (SLD) affects a consistent percentage of school-aged children. It is estimated that 5–15% of school-age children across different languages and cultures have an SLD [1]. SLDs are neurobiologically determined by an interaction of genetic and environmental factors [2], reducing the ability of the brain to efficiently and accurately perceive or process verbal or non-verbal information. SLD cannot be explained by factors such as intellectual disabilities, vision deficits, hearing impairments, other mental or neurological disorders, adverse psychosocial circumstances, insufficient proficiency in the language, or inadequate educational methods and cannot be attributed to emotional disturbances, cultural differences, or disadvantages [3,4].

According to the fifth edition of the Diagnostic and Statistical Manual of Mental Disorders (DSM-5) [1], SLD is characterized by the following diagnostic criteria: difficulties in learning and academic skills, as indicated by the presence of at least one of the following symptoms, that have persisted for at least 6 months despite the provision of interventions targeting those difficulties: (1) inaccurate or slow and effortful word reading; (2) difficulty in the comprehension of the meaning of what is read; (3) difficulties with spelling; (4) difficulties with written expression; (5) difficulties mastering number sense, number facts, or calculation; (6) difficulties with mathematical reasoning.

Specific impairments included in SLDs are the following:

(1) Impairment in reading: This includes difficulties in word reading accuracy, reading rate or fluency, and reading comprehension. The term “dyslexia” is used to describe a pattern of learning difficulties where individuals struggle with accurate or fluent word recognition, have poor decoding and spelling abilities but exhibit normal IQ, receive appropriate teaching and environmental support, do not have sensory deficits, and show relative resistance to treatment [5,6].
(2) Impairment in written expression: SLDs related to impaired written expression are categorized into two groups: one involving difficulties with spelling accuracy, punctuation accuracy, and grammar accuracy, and the other involving difficulties with organization or clarity of written expression [7]. The term “dysgraphia” is used to describe a pattern of difficulties where individuals exhibit distorted writing despite receiving thorough instruction.
(3) Impairment in mathematics: This encompasses difficulties in number sense, memorization of arithmetic facts, accurate or fluent calculation, and mathematical reasoning. The term “dyscalculia” is used to describe a pattern of difficulties characterized by deficits in processing numerical information, learning arithmetic facts, and performing accurate or fluent calculations.

Although the majority of SLDs occur in the reading domain, children diagnosed with this disorder can underperform in writing and mathematics as well [8]. The comorbidity between the SLD domains is frequent, especially between reading and math. The affected academic skills are substantially and quantifiably below those expected for the individual’s chronological age and they lead to significant impairment in academic or occupational performance and daily life activities. Moreover, a link between SLD and emotional problems has been repeatedly reported in psychological literature. According to recent studies, children with SLD experience significantly more emotional distress in comparison to their peers without it [9,10,11,12,13,14,15,16,17]. Furthermore, children with SLD have higher rates of school dropout and lower college attendance than those without SLD [18,19]; School dropout and co-occurring depressive symptoms result in an increased risk for mental health outcomes, including suicidality. Challenges arise during the period of school-age development, although they may not become fully apparent until the demands placed on individuals with affected academic skills surpass their capacities. SLD can also have implications for adult life, potentially leading to detrimental effects on both physical and mental well-being [20].

The present study aims to investigate the profile of children and adolescents with SLD. Specifically, the study seeks to determine whether children and adolescents with SLD associated with behavioral symptoms exhibit significantly different characteristics compared to children with a typical SLD profile, thus representing a distinct profile. The study also intends to investigate correlations between cognitive and behavioral measures in both profiles. Furthermore, the study aims to identify the skills that are most significantly compromised during the initial evaluation (T0) and measure the potential improvement of these impairments following treatment. Differences between children and adolescents due to gender are tested as well. On the basis of these analyses, the study aims to suggest effective strategies for prevention and intervention in SLD.

## 2. Methods

### 2.1. Participants

Participants in the study were recruited at the “Fondazione Policlinico A. Gemelli”. It is based in Rome and serves as a specialized center in Italy for diagnosing SLD. In this institution, children and adolescents presenting with potential learning disorders undergo a thorough assessment involving comprehensive evaluations of cognitive abilities, specific learning skills, and emotional-behavioral functioning, following the guidelines outlined by the World Health Organization (WHO). These assessments aim to provide a comprehensive understanding of the learning difficulties, enabling the development of tailored interventions and support strategies to address their unique needs [21].

A total of 2.500 children and adolescents were evaluated for this prospective study from 2016 to 2020. Only 191 children met the inclusion criteria, which were the following: (1) children and adolescents diagnosed with SLD; (2) primary school age at first evaluation (T0) = 8 ± 2 years; (3) follow-up (T1) after at least 3 years from T0; (4) absence of cognitive impairment (IQ > 85) and associated neuropsychiatric disorders or additional neurosensory deficits (hearing or vision problems). The final sample consisted of 108 males and 83 females, 144 children, and 47 adolescents. The mean age of the sample was 10.4 years.

At the initial assessment (T0), a comprehensive evaluation was conducted, encompassing cognitive, academic, emotional, and behavioral aspects (see Procedure). Based on the assessment results, the sample was divided into two groups: One group comprised children with a typical SLD profile (N = 103), while the other group consisted of children exhibiting atypical SLD with emotional-behavioral impairments (N = 88). Subsequently, all participants underwent a follow-up assessment (T1) after a minimum interval of 3 years from T0. During this period, children and adolescents received psychotherapy and speech therapy interventions. Both groups were subject to the same assessment protocol at T1, allowing for the evaluation of any changes or progress over time. Psychotherapy and speech therapy were determined according to the patient’s needs. Psychotherapy was conducted by agreeing with the families on the objectives to improve emotional and behavioral aspects. Speech therapy was carried out with tailored approaches defined on the basis of the patient’s needs, with particular attention to the implementation of working memory and processing capacity.

### 2.2. Procedure

The cognitive profile was assessed using the Wechsler Intelligence Scale, fourth edition (WISC-IV) [22]. WISC-IV was used to estimate general intellectual functioning (IQ) and provide information on specific aspects of cognitive functioning. The scale is divided into ten subtests that contribute to a full-scale IQ (FIQ). A total of four composite scores can be obtained from the 10 subtests: Verbal Comprehension Index (VCI), an overall measure of verbal concept formation; Visuospatial Index (VSI), a measure of visuoperceptual organization and non-verbal reasoning; Working Memory Index (WMI), a measure of memory span and freedom from distractibility; Processing Speed Index (PSI), a measure of fast visuomotor integration and learning [23].

To evaluate academic skills, the following tests were administered:

- MT-2 reading battery, a standardized set of tests assessing speed and accuracy in reading and text comprehension abilities [24,25];
- BVSCO-2, a standardized test assessing writing skills, more specifically orthographic competence [26];
- AC-MT 6–11, an arithmetic standardized test assessing calculation skills and mathematical reasoning [27].

To assess behavioral and emotional impairments, the Child Behavior Checklist (CBCL) 6–18 was utilized. The CBCL is a structured rating scale completed by caregivers (typically parents) to evaluate their child’s social and emotional difficulties within the past 6 months or at the current time. It comprises 113 items that pertain to behavioral and emotional problems. Parents rate each item using a response set of 0 (not true), 1 (sometimes true), or 2 (very true). The scale is designed for children and adolescents between the ages of 6 and 18 years. The CBCL yields various scores, including a total score. Factor analytic studies have identified two broad-band factors known as internalizing and externalizing. Furthermore, there are eight narrow-band syndrome scales: anxious/depressed, withdrawn/depressed, somatic complaints (these three scales contribute to the internalizing factor), social problems, thought problems, attention problems, rule-breaking behavior, and aggressive behavior (the latter two scales contribute to the externalizing factor) [28,29,30]. To define a behavioral/emotional impairment, cutoff scores were considered for the three scales (internalizing, externalizing, and total) and individual subscales.

Both at T0 and T1, participants underwent various assessments during two sessions at Fondazione Policlinico Gemelli. In the first session, WISC-IV was administered, and a parent was requested to complete CBCL. In the second session, tests were administered to explore reading and writing abilities, text comprehension, and mathematical calculation skills.

## 3. Results

### 3.1. Descriptive Data

#### 3.1.1. Total Sample at T0

Analyses conducted on the total sample (children + adolescents with typical SLD and children + adolescents with atypical SLD) about cognitive evaluation at T0 showed that 12% (24/191) of the participants had an impairment (borderline or below average scores) in VCI, 14% (28/191) in VSI, 52% (101/191) in WMI, and 37% (71/191) in PSI.

Analysis of the total sample academic skills at T0 revealed that 81% of the participants (156/191) showed an impairment in writing accuracy, 56% (108/191) in reading accuracy, 50% (96/191) in text comprehension, 53% (103/191) in reading speed, 41% (79/191 in calculation, and 38% (74/191) in number comprehension.

Regarding the behavioral and emotional evaluation of the total sample at T0, the analysis indicated that 52% (100/191) showed an impairment condition on the internalizing scale (anxious/depression and withdrawn/depressed are the most compromised scales), 40% (77/191), 24% (47/191) in externalizing scale, and 40% (77/191) in the total problems scale.

#### 3.1.2. Typical SLD Sample at T0

SLD typical sample’s cognitive assessment at T0 showed that 13% (14/103) had an impairment (borderline or below average scores) in VCI, 16% (17/103) in PSI, 56% (58/103) in WMI, and 36% (37/103) in PSI.

The analysis of the academic performance of the typical sample at T0 resulted in 83% (86/103) showing impairment in writing accuracy, 39% (40/103) in reading accuracy, 63% (65/103) in text comprehension, 60% (62/103) in reading speed, 47% (48/103) in calculation, and 32% (33/103) in math reasoning.

As regards the emotional and behavioral evaluation of the typical SLD sample at T0, 16% (17/103) presented an impairment in the internalizing scale, 4% (4/103) an impairment in the externalizing scale, and 5% (5/103) an impaired condition in the total problems scale.

#### 3.1.3. Atypical SLD Sample at T0

On analyzing the SLD atypical sample’s cognitive evaluation at T0, it was observed that 12% (10/86) exhibited an impairment (borderline or below average scores) in VCI, 13% (11/86) in VSI, 49% (42/86) in WMI, and 39% (34/86) in PSI.

Academic performance analysis at T0 revealed 79% (68/86) having an impairment in spelling accuracy, 44% (38/86) in reading accuracy, 51% (44/86) in text comprehension, 48% (41/86) in reading speed, 55% (47/86) in calculation skill, and 44% (38/86) in number comprehension.

Analyzing SLD atypical sample’s emotional and behavioral evaluation at T0, it was found that 94% (81/86) showed impairment on the internalizing scale, 49% (42/86) on the externalizing scale, and 81% (70/86) on the total problems scale. Two children were not included in the samples since they presented several other comorbidities.

#### 3.1.4. Comparison of the Two Samples at T0

**Figure 1:**
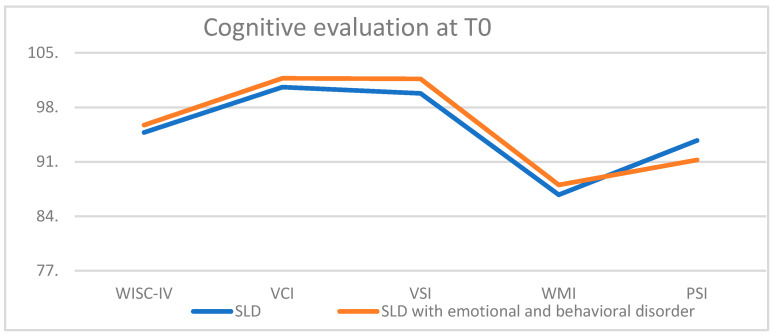
Cognitive evaluation at T0.

From the comparison of the cognitive evaluation at T0 between the SLD typical and SLD atypical samples, no differences between specific aspects of cognitive functioning (VCI, VSI, WMI, and PSI) were observed (Figure 1).

**Figure 2:**
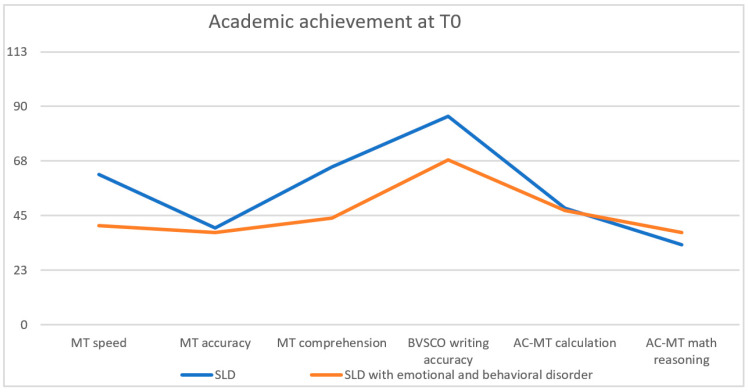
Academic achievement at T0.

Comparing the academic skills between the SLD typical and the SLD atypical sample at T0 (Figure 2), a difference was detected, with SLD typical children and adolescents showing a greater impairment in writing accuracy, text comprehension, and reading speed; SLD atypical children and adolescents showed increased difficulties in reading accuracy and number comprehension.

**Figure 3:**
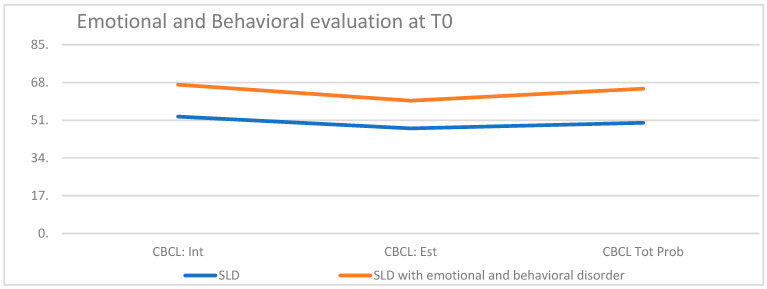
Emotional and behavioral evaluation at T0.

CBCL scores at T0 were higher in children and adolescents with atypical SLD compared to those characterized by typical SLD, indicating a more compromised profile regarding emotional and behavioral components (Figure 3).

#### 3.1.5. Total Sample at T1

Total sample’s (both children and adolescents with typical and atypical SLD profiles) cognitive evaluation at T1 revealed that 13% (24/191) showed an impairment (borderline or below average scores) in VCI, 12% (23/191) in VSI, 47% (91/191) in WMI, and 32% (62/191) in PSI.

Academic performance analysis at T1 resulted in 73% (140/191) with impaired writing accuracy, 33% (64/191) impaired reading accuracy, 54% (104/191) impaired text comprehension, 53% (103/191) impaired reading speed, 56% (107/191) impaired calculation, and 40% (78/191) impaired math reasoning.

Regarding the total sample’s emotional and behavioral evaluation at T1, 52% (101/191) displayed problems on the Internalizing Scale, while an impairment in the externalizing scale was found in 18% (35/191) of the sample (attention problems and thought problems were the most compromised scales), and 37% (71/191) showed lower scores at the total problems scale.

#### 3.1.6. Typical SLD Sample at T1

Cognitive performance assessment in the typical SLD sample at T1 suggested 13% (14/103) presenting an impairment (borderline or below average scores) in VCI, 12% (10/86) in VSI, 49% (51/103) in WMI, and 36% (37/103) in PSI.

Regarding academic skills evaluation at T1 in this group, the analysis suggested that 76% (78/103) exhibited an impairment in writing accuracy, 30% (31/103) in reading accuracy, 52% (54/103) in text comprehension, 54% (56/103) in reading speed, 52% (54/103) in calculation, and 38% (39/103) in math reasoning.

Disordered emotional and behavioral components at T1 were observed in 17% (18/103) of the internalizing scale, while impairment in the externalizing scale was found in 1% (1/103) of the typical sample and 4% (4/103) for the total problems scale.

#### 3.1.7. Atypical SLD Sample at T1

The SLD atypical sample’s cognitive performance analysis was performed at T1, with 12% (10/86) showing an impairment (borderline or below average scores) in VCI, 13% (11/86) in VSI, 42% (36/86) in WMI, and 12% (10/86) in VCI.

Data obtained from the academic skills evaluation in this sample also demonstrated that 64% (55/86) featured problems in writing accuracy, 34% (29/86) in reading accuracy, 46% (40/86) in reading speed, 50% (43/86) in text comprehension, 55% (47/86) in calculation, and 41% (35/86) in math reasoning.

CBCL scores at T1 in this sample suggested disturbances in emotional and behavioral components for 89% (77/86) on the internalizing scale, 39% (34/86) on the externalizing scale, and 71% (61/86) on the total problems scale. Two children were not included in the samples because they had an atypical profile.

#### 3.1.8. Comparison of the Two Samples at T1

**Figure 4:**
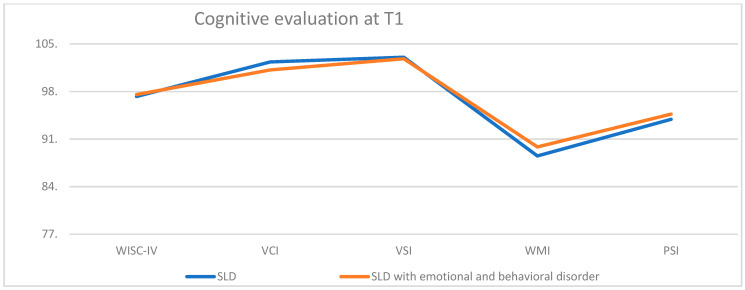
Cognitive evaluation at T1.

From the comparison of the cognitive evaluation at T1 between the typical and atypical SLD samples (Figure 4), no significant difference between specific aspects of cognitive functioning (VCI, VSI, WMI, and PSI) was found.

**Figure 5:**
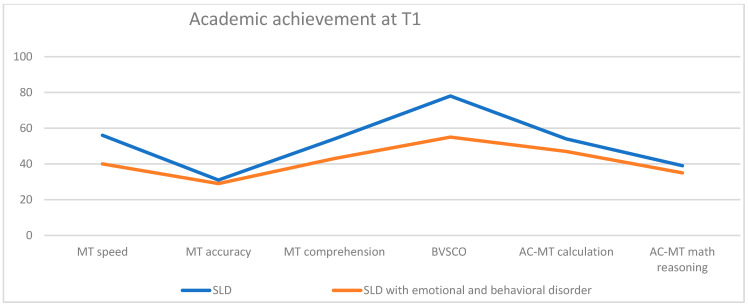
Academic achievement at T1.

By comparing the academic abilities assessment at T1 between the two groups (Figure 5), a difference was found, with children and adolescents with a typical SLD profile showing greater impairment in writing accuracy, reading speed, text comprehension, and calculation compared to the atypical SLD sample, whose abilities were less compromised.

By contrast, based on CBCL score comparisons, children and adolescents characterized by an atypical SLD pattern showed more impaired emotional and behavioral aspects compared to the typical SLD group. The percentages of impaired cognitive, academic, and emotional-behavioral skills at T0 and T1 for both groups are presented in Table 1.

### 3.2. Statistical Analyses

#### 3.2.1. Gender Differences

A significant difference in the mean scores of FIQ obtained at T0 and T1 between males and females emerged (F(1, 177) = 5.27, *p* = 0.023, η² = 0.029). Specifically, the mean score for males at T0 was 93.1 (SD = 12.3), while at T1 they achieved a mean score of 96.7 (SD = 12.8). The mean score for females at T0 was 97.8 (SD = 12.8), whereas at T1 they obtained a mean score of 97.1 (SD = 13.6). A significant improvement in scores from T0 to T1 was observed in the male sample compared to the female samples, who initially had a significantly higher score at T0 but showed a slight decline at T1.

#### 3.2.2. Children vs. Adolescents

A significant difference in the mean scores of FIQ obtained at T0 and T1 between children and adolescents was found (F(1, 178) = 4.202, *p* = 0.042, η² = 0.023). Specifically, the mean score for children at T0 was 95.0 (SD = 12.0), while at T1 they achieved a score of 97.0 (SD = 12.8). Therefore, an improvement in performance was observed, which was not observed in adolescents. A significant difference in the mean scores of writing skills obtained at T0 and T1 between children and adolescents (F(1, 178) = 15.87, *p* < 0.001, η² = 0.126) emerged as well. In this case, the mean score for children at T0 was 1.77 (SD = 0.94), while at T1 they obtained a score of 1.30 (SD = 0.63). The mean score for adolescents at T0 was 2.05 (SD = 1.06), whereas at T1 they achieved a score of 2.26 (SD = 1.04). Therefore, a slight decline in scores from T0 to T1 was observed in the children group compared to the adolescent group, which initially had a significantly higher score than the children and showed a similarly significant improvement in performance at T1.

#### 3.2.3. Correlations Patterns

*Typical SLD sample*: In the typical SLD sample at T0, significant correlations emerged between number comprehension and calculation and VCI. As regards behavioral aspects, a positive correlation between the internalizing scale and the accuracy of writing was found. Furthermore, a relationship emerged between the combination of the different emotional and behavioral aspects (internalizing, externalizing, and total problems) and academic skills. At T1 associations between VCI and WMI and mathematical skills emerged and the correlation between internal aspects and accuracy of writing was statistically significant. All correlations are reported in Table 2 and Table 3.

*Atypical SLD Sample*: At T0, a significant correlation emerged between reading speed and text comprehension (r = 0.626, *p* < 0.01), VSI, (r = 0.365, *p* < 0.01), WMI (r = 0.352, *p* < 0.01), and PSI (r = 0.250, *p* < 0.05), as well as significant positive correlations were evident between the internalizing, externalizing, and total problems scales and academic skills, (*p* < 0.01). T1 confirms the influence of specific components of cognitive functioning on academic skills. All correlations are presented in Table 4 and Table 5.

#### 3.2.4. Differences between T0 and T1

*Total Sample*: *t* tests revealed a significant increase in FIQ scores (t (190) = −2.544, *p* = 0.012). Specifically, the scale score increased from 95.23 (T0) to 96.86 (T1). Additionally, a significant increase in VSI scores was observed (t (190) = −2.173, *p* = 0.031). Specifically, the scale score increased from 100.92 (T0) to 102.63 (T1). Analyzing the CBCL scores, a significant decrease in total problems scale scores was observed (t (190) = 2.225, *p* = 0.027): The score decreased from 57.00 (T0) to 55.62 (T1). Similarly, the ANOVA showed that there was a significant difference between T0 and T1 in the FIQ scores (F (1; 190) = 6.474, *p* = 0.012; η² = 0.033) and in the VSI as well (F (1; 190) = 4.724, *p* = 0.031; η² = 0.024). The significant difference between T0 and T1 in the CBCL total problems scale scores was confirmed by ANOVA (F (1; 190) = 4.952, *p* = 0.027; η² = 0.027).

*Typical SLD Sample*: Analyzing the trend between the typical SLD sample at T0 and T1, no statistically significant improvement was observed in general cognitive functioning, academic abilities, or emotional and behavioral aspects.

*Atypical SLD Sample:* Investigating the trend of the atypical SLD sample between T0 and T1, no significant improvement has been detected in general cognitive functioning, academic achievement, and emotional and behavioral aspects.

## 4. Discussion and Conclusions

This study investigated cognitive, academic, emotional, and behavioral abilities in children and adolescents diagnosed only with SLD (typical SLD) and in children and adolescents diagnosed with SLD associated with an emotional and behavioral impairment (atypical SLD). In this way, it was possible to better define a complete phenotype for both cases. According to the literature, children with SLD report higher levels of emotional disturbances compared to their peers without learning disabilities, specifically anxious symptomatology. Furthermore, to analyze the evolution of cognitive, academic, emotional, and behavioral profiles over time in the typical and atypical SLD samples, a follow-up (T1) was carried out after at least three years from the first evaluation (T0). In the period between T0 and T1, all the participants received psychotherapy and speech therapy.

The results indicated that the most compromised cognitive ability in SLD was working memory. Considering the total sample, 101 out of 191 participants showed a below-average working memory index. This result emerged from the entire sample (typical + atypical SLD) at T0 and follow-up. This finding confirms previous evidence highlighting working memory as a predictor of general cognitive functioning and learning processes [31]. Furthermore, the working memory index demonstrated correlations in the typical sample of SLD with the verbal comprehension index, as well as number comprehension and calculation. These findings support previous studies that suggest a significant role for working memory in the acquisition of mathematical abilities. Literature evidence indicates that a specific impairment in the functioning of the visuospatial sketchpad is a characteristic deficit pattern observed in children with specific arithmetic skill disorders [32]. Additionally, the central executive function also appears to be notably impaired [33]. However, there is inconsistency in the data regarding which specific domains of working memory are involved in mathematical abilities. While Passolunghi [34] found that all three working memory domains play a role in mathematical abilities, specific abnormalities in working memory domains remain unclear and there is contradictory evidence regarding the involvement of the phonological loop and visuospatial sketchpad. Therefore, future studies should explore new perspectives by investigating the specific working memory domains implicated in each academic impairment. In contrast to working memory, the least affected cognitive skills were the verbal comprehension index and the visuospatial index.

Regarding academic abilities, writing skills were the most compromised across all samples. Text comprehension and reading speed were also impaired, although to a lesser extent compared to writing skills. As previously mentioned, working memory plays a crucial role in the normal development of academic skills. Similarly, when compromised, it can lead to deficits in the writing domain as well [35]. It is reported that between 10% and 30% of children have difficulties in writing [36]. Moreover, dysgraphia seems more common in boys than in girls [37]. In line with these findings, in our study writing skills were the most compromised in all samples at T0 as well as at follow-up.

Concerning the behavioral analysis, results showed that the internalizing scale was the most impaired in the typical and atypical SLD samples. Once more, our findings appear in line with those from previous studies, indicating that children with SLD experience significantly higher anxiety levels compared to controls, as well as increased anxiety and depression levels [38,39]. According to these results, learning difficulties negatively impact the psychological wellness of children and adolescents with a diagnosis of SLD.

A relationship emerged between gender and emotional and behavioral impairment in the total sample. In detail, males tended to adopt more externalizing behavior than females.

The comparison between typical and atypical SLD samples did not show significant differences in terms of cognitive functions, indicating that the working memory index is the most compromised in both samples. Thus, although the emotional and behavioral components differentiated the two samples, they both exhibited a similar picture in terms of cognitive functions. However, with regards to academic skills, even if not significant, subjects in the typical SLD sample showed greater impairment in reading speed, text comprehension, writing accuracy, and arithmetic areas compared to the atypical SLD sample subjects.

Further statistical analyses in the SLD sample at T0 indicated a significant correlation between arithmetic skills and the total IQ and between the verbal comprehension index and the working memory index. A significant correlation between internalizing scale and writing skills was detected. The statistical analysis conducted on the same sample at T1 showed a significant correlation between total IQ, text comprehension, and arithmetic skills. Furthermore, the working memory index appeared to be significantly correlated with writing and arithmetic abilities. For these subjects, as noted at T0, the significant correlation between the internalizing scale and writing abilities has been confirmed.

A longitudinal analysis of the total sample showed improvements in some cognitive abilities from T0 to T1, such as FIQ and VSI. Likewise, this analysis showed improvement from an emotional and behavioral perspective, specifically in the total problems scale.

In summary, the data obtained from the two samples provided a similar picture. Particularly at the first evaluation, both samples reported working memory to be the most compromised, along with writing abilities being the most impaired in terms of academic skills. In addition, high levels of impairment emerged in text comprehension, reading speed, and calculation. These results are in line with previous studies showing a link between working memory impairment and mathematical and reading disabilities [40,41,42,43]. The second evaluation confirmed these findings, with working memory being greatly impaired as well as writing ability. Reading speed, text comprehension, and calculation also showed high levels of impairment. Focusing on the comparison between the two samples at T0 and T1, no significant improvements in any cognitive function, academic skills, and behavioral scale were found. However, both samples improved slightly in working memory: from 56% at T0 to 49% at T1 for the typical SLD sample and from 49% at T0 to 42% at T1 for the atypical SLD sample. Results also indicated a slight improvement in writing skills as compared to academic skills, from 83% to 76% at T0 for the typical SLD sample and from 79% at T0 to 64% at T1 for the atypical one. Results of the SLD sample characterized by behavioral impairment showed a slight improvement—although not significant—in all behavioral scales, while, on the other hand, the SLD sample developed a slight worsening in the internalizing scale and a minor improvement relating to the externalizing and total problems scales. These findings may suggest that psychotherapy and speech therapy may not directly lead to a significant improvement in terms of general cognitive functioning and academic skills. To reach this specific goal, more specific cognitive and working memory training could be implemented to better impact cognitive abilities as well as academic skills and emotional domains. In a similar framework, some authors proposed different strategies to treat SLD: specific assistance targeting academic skills, on the one hand, and psychotherapeutic treatment aimed at handling the related psychological symptoms, on the other.

It will be interesting to study whether these results will be confirmed in the future, considering the post-pandemic period. Following the COVID-19 pandemic, the psychological implications for kids have increased. More research is needed to better specify the correlations between emotional aspects and learning difficulties following the pandemic.

## Figures and Tables

**Table 1:** Percentage of impairment pre-treatment (T0) and post-treatment (T1) in cognitive and academic skills and emotional-behavioral aspects in typical and atypical samples.

Test	Typical SLD		SLD + Behavioral/Emotional Impairments	
	T0	T1	T0	T1
*Cognitive measures*				
WISC-IV				
VCI	13%	12%	12%	12%
VSI	16%	13%	13%	13%
WMI	56%	49%	49%	42%
PSI	36%	36%	39%	27%
MT-2, BVSCO-2, AC-MT				
Writing accuracy	83%	76%	79%	64%
Reading accuracy	39%	30%	44%	34%
Text comprehension	63%	52%	51%	50%
Reading speed	60%	54%	48%	46%
Calculation	47%	52%	55%	55%
Math reasoning	32%	38%	0%	41%
CBCL				
Internalizing Scale	16%	17%	94%	89%
External Scale	4%	4%	49%	39%
Total Problems Scale	5%	1%	81%	71%

**Table 2:** Pearson’s correlation coefficients regarding WISC-IV, academic measures, and CBCL, pre-treatment outcomes.

Typical Sample (T0)						
	MT Speed	MT Accuracy	Text Comprehension	BVSCO Accuracy	AC-MT Calculation	AC-MT Reasoning
IQ	−0.089	0.139	0.176	0.010	0.378 **	0.296 *
VCI	0.010	0.085	0.096	−0.048	0.385**	0.378 **
VSI	−0.079	0.148	0.191	0.154	0.174	0.150
WMI	0.118	0.133	0.080	0.045	0.398 **	0.010
PSI	0.054	0.133	0.053	−0.072	0.199	0.231
Internalizing	−0.077	−0.41	−0.002	−0.146 *	0.102	0.137
Externalizing	−0.086	0.050	−0.102	−0.066	0.069	0.019
Total problems	0.034	0.100	−0.060	−0.101	0.067	0.031

** *p* < 0.01; * *p* < 0.05.

**Table 3:** Pearson’s correlation coefficients regarding WISC-IV, academic measures, and CBCL, post-treatment outcomes.

Typical Sample (T1)						
	MT Speed	MT Accuracy	Text Comprehension	BVSCO Accuracy	AC-MT Calculation	AC-MT Reasoning
IQ	0.086	−0.055	0.323 **	0.023	0.334 **	0.451 **
VCI	0.087	−0.018	0.164	−0.010	0.195	0.341 **
VSI	0.079	0.63	0.304 **	0.150	0.209 *	0.300 **
WMI	0.103	0.008	0.127	0.295 **	0.277 **	0.235 *
PSI	0.163	0.052	0.111	0.102	0.151	0.205
Internalizing	0.073	−0.063	−0.145	−0.204 *	0.013	0.038
Externalizing	−0.093	−0.059	0.142	0.090	0.040	0.095
Total problems	−0.014	−0.120	−0.129	0.063	0.067	0.079

** *p* < 0.01; * *p* < 0.05.

**Table 4:** Pearson’s correlation coefficients regarding WISC-IV, academic measures, and CBCL, pre-treatment outcomes.

Atypical Sample (T0)						
	MT Speed	MT Accuracy	Text Comprehension	BVSCO Accuracy	AC-MT Calculation	AC-MT Reasoning
IQ	0.292 **	0.326 **	0.256 *	0.050	0.190	0.162
VCI	0.030	0.006	0.052	−0.035	0.028	0.048
VSI	0.372 **	0.500 **	0.130	0.154	−0.182	0.163
WMI	0.091	0.165	−0.062	0.218 *	0.106	0.139
PSI	0.372 **	0.500 **	0.130	0.154	−0.061	−0.129
Internalizing	−0.053	−0.049	0.069	0.027	0.172	0.044
Externalizing	0.178	0.194	0.167	−0.060	−0.028	0.064
Total problems	0.180	0.178	0.164	−0.036	−0.066	−0.025

** *p* < 0.01; * *p* < 0.05.

**Table 5:** Pearson’s correlation coefficients regarding WISC-IV, academic measures, and CBCL post-treatment outcomes.

Atypical Sample (T1)						
	MT Speed	MT Accuracy	Text Comprehension	BVSCO Accuracy	AC-MT Calculation	AC-MT Reasoning
IQ	0.065	0.080	0.215 *	0.108	0.239 *	0.218
VCI	−0.046	−0.017	0.210	0.094	0.089	0.172
VSI	0.089	0.046	0.038	0.168	−0.014	0.052
WMI	0.132	−0.115	0.236 *	0.205	0.286 *	0.136
PSI	0.047	−0.001	0.013	0.122	0.001	0.054
Internalizing	0.120	0.015	0.048	0.122	−0.018	−0.076
Externalizing	0.179	0.184	0.046	0.046	0.011	−0.014
Total problems	0.151	0.139	0.057	0.143	0.092	0.157

* *p* < 0.05.

## Data Availability

Data sharing is not applicable to this article.

## References

[1] American Psychiatric Association (2013). Diagnostic and Statistical Manual of Mental Disorders.

[2] Xia Z., Hancock R., Hoeft F. (2017). Neurobiological bases of reading disorder Part I: Etiological investigations. Lang. Linguist. Compass.

[3] Al-Mahrezi A., Al-Futaisi A., Al-Mamari W. (2016). Learning Disabilities: Opportunities and challenges in Oman. Sultan Qaboos Univ. Med. J..

[4] Alloway T.P. (2009). Working memory, but not IQ, predicts subsequent learning in children with learning difficulties. Eur. J. Psych. Assess..

[5] Kohli A., Sharma S., Padhy S.K. (2018). Specific learning disabilities: Issues that remain unanswered. Ind. J. Psychol. Med..

[6] Döhla D., Heim S. (2015). Developmental dyslexia and dysgraphia: What can we learn from the one about the other?. Front. Psychol..

[7] McCloskey M., Rapp B. (2017). Developmental dysgraphia: An overview and framework for research. Cogn. Neuropsychol..

[8] Haft S.L., Duong P.H., Ho T.C., Hendren R.L., Hoeft F. (2019). Anxiety and attentional bias in children with specific learning disorders. J. Abnorm. Child Psychol..

[9] Abrams J.C. (1986). On learning disabilities: Affective considerations. J. Read. Writ. Learn. Disabil. Int..

[10] Beitchman J.H., Young A.R. (1997). Learning disorders with a special emphasis on reading disorders: A review of the past 10 years. J. Am. Acad. Child Adolesc. Psychiatry.

[11] Bender W.N., Wall M.E. (1994). Social-emotional development of students with learning disabilities. Learn. Disabil. Q..

[12] Bryan T., Bryan J.H. (1977). The social-emotional side of learning disabilities. Behav. Dis..

[13] Bryan T., Burstein K., Ergul C. (2004). The social-emotional side of learning disabilities: A science-based presentation of the state of the art. Learn. Disabil. Q..

[14] Cohen J. (1986). Learning disabilities and psychological development in childhood and adolescence. Ann. Dyslexia.

[15] Elksnin L.K., Elksnin N. (2004). The social-emotional side of learning disabilities. Learn. Disabil. Q..

[16] Rock E.E., Fessler M.A., Church R.P. (1997). The concomitance of learning disabilities and emotional/behavioral disorders: A conceptual model. J. Learn. Dis..

[17] Spreen O. (1989). The relationship between learning disability, emotional disorders, and neuropsychology: Some results and observations. J. Clin. Exp. Neuropsychol..

[18] Morrison G.M., Cosden M.A. (1997). Risk, resilience, and adjustment of individuals with learning disabilities. Learn. Disabil. Q..

[19] Murray C., Goldstein D.E., Nourse S., Edgar E. (2000). The postsecondary school attendance and completion rates of high school graduates with learning disabilities. Learn. Dis. Res. Pract..

[20] McDowell M. (2018). Specific learning disability. J. Paediatr. Child Health.

[21] World Health Organization (2019). International Statistical Classification of Diseases and Related Health Problems.

[22] Wechsler D., Orsini A. (2003). Wechsler Intelligence Scale for Children.

[23] Orsini A., Pezzuti L., Picone L. (2012). WISC-IV. Contributo alla Taratura Italiana.

[24] Cornoldi C., Colpo G., Gruppo M.T. (2011). MT Reading Test.

[25] Sartori G., Job R., Tressoldi P.E. (2007). DDE-2: Batteria per la Valutazione della Dislessia e della Disortografia Evolutiva-2: Protocollo di Registrazione.

[26] Tressoldi P.E., Cornoldi C., Re A.M. (2013). BVSCO-2. Batteria per la Valutazione della Scrittura e della Competenza Ortografica-2.

[27] Cornoldi C., Lucangeli D., Bellina M. (2012). Test AC-MT 6-11—Test di Valutazione delle Abilità di Calcolo e Soluzione di Problemi.

[28] Achenbach T.M. (1991). Manual for the Teacher’s Report Form and 1991 Profile.

[29] Achenbach T.M., Edelbrock C. (1986). Manual for the Teacher’s Report Form and Teacher Version of the Child Behavior Profile.

[30] Handwerk M.L., Marshall R.M. (1998). Behavioral and emotional problems of students with learning disabilities, serious emotional disturbance, or both conditions. J. Learn. Dis..

[31] Maehler C., Schuchardt K. (2016). The importance of working memory for school achievement in primary school children with intellectual or learning disabilities. Res. Dev. Disabil..

[32] Schuchardt K., Maehler C., Hasselhorn M. (2008). Working memory deficits in children with specific learning disorders. J. Learn. Dis..

[33] Siegel L.S., Ryan E.B. (1989). The development of working memory in normally achieving and subtypes of learning disabled children. Child Dev..

[34] Passolunghi M.C., Siegel L.S. (2001). Short-term memory, working memory, and inhibitory control in children with specific arithmetic learning disabilities. J. Exp. Child Psychol..

[35] Berninger V.W., Mody M., Silliman E.R. (2008). Defining and Differentiating Dysgraphia, Dyslexia, and Language Learning Disability within a Working Memory Model. Brain, Behavior, and Learning in Language and Reading Disorders.

[36] Kushki A., Schwellnus H., Ilyas F., Chau T. (2011). Changes in kinetics and kinematics of handwriting during a prolonged writing task in children with and without dysgraphia. Res. Dev. Disabil..

[37] Berninger V.W., May M.O. (2011). Evidence-based diagnosis and treatment for specific learning disabilities involving impairments in written and/or oral language. J. Learn. Disabil..

[38] Nelson J.M., Harwood H. (2011). Learning disabilities and anxiety: A meta-analysis. J. Learn. Dis..

[39] Nelson J.M., Liebel S.W. (2018). Socially desirable responding and college students with dyslexia: Implications for the assessment of anxiety and depression. Dyslexia.

[40] Swanson H.L. (1994). Short-term memory and working memory: Do both contribute to our understanding of academic achievement in children and adults with learning disabilities?. J. Learn. Dis..

[41] Swanson H.L. (1999). What develops in working memory? A life span perspective. Dev. Psychol..

[42] Swanson H.L., Ashbaker M.H., Lee C. (1996). Learning disabled readers working memory as a function of processing demands. J. Exp. Child Psychol..

[43] Swanson H.L., Saez L., Swanson H.L., Graham S., Harris K.R. (2003). Memory difficulties in children and adults with learning disabilities. Handbook of Learning Disabilities.

